# Genetic Association Study of Common Mitochondrial Variants on Body Fat Mass

**DOI:** 10.1371/journal.pone.0021595

**Published:** 2011-06-29

**Authors:** Tie-Lin Yang, Yan Guo, Hui Shen, Shu-Feng Lei, Yong-Jun Liu, Jian Li, Yao-Zhong Liu, Na Yu, Jia Chen, Ting Xu, Yu Cheng, Qing Tian, Ping Yu, Christopher J. Papasian, Hong-Wen Deng

**Affiliations:** 1 Key Laboratory of Biomedical Information Engineering of Ministry of Education, and Institute of Molecular Genetics, School of Life Science and Technology, Xi'an Jiaotong University, Xi'an, Shaanxi, People's Republic of China; 2 School of Public Health and Tropical Medicine, Tulane University, New Orleans, Louisiana, United States of America; 3 Institute of Bioscience and Biotechnology, School of Science, Beijing Jiaotong University, Beijing, People's Republic of China; University of Bristol, United Kingdom

## Abstract

Mitochondria play a central role in ATP production and energy metabolism. Previous studies suggest that common variants in mtDNA are associated with several common complex diseases, including obesity. To test the hypothesis that common mtDNA variants influence obesity-related phenotypes, including BMI and body fat mass, we genotyped a total of 445 mtSNPs across the whole mitochondrial genome in a large sample of 2,286 unrelated Caucasian subjects. 72 of these 445 mtSNPs passed quality control criteria, and were used for subsequent analyses. We also classified all subjects into nine common European haplogroups. Association analyses were conducted for both BMI and body fat mass with single mtSNPs and mtDNA haplogroups. Two mtSNPs, *mt4823* and *mt8873* were detected to be significantly associated with body fat mass, with adjusted *P* values of 4.94×10^-3^ and 4.58×10^-2^, respectively. The minor alleles *mt4823* C and *mt8873* A were associated with reduced fat mass values and the effect size (β) was estimated to be 3.52 and 3.18, respectively. These two mtSNPs also achieved nominally significant levels for association with BMI. For haplogroup analyses, we found that haplogroup X was strongly associated with both BMI (adjusted *P* = 8.31×10^-3^) and body fat mass (adjusted *P* = 5.67×10^-4^) Subjects classified as haplogroup X had lower BMI and fat mass values, with the β estimated to be 2.86 and 6.03, respectively. Our findings suggest that common variants in mitochondria might play a role in variations of body fat mass. Further molecular and functional studies will be needed to clarify the potential mechanism.

## Introduction

All nucleated cells contain mitochondria, organelles whose most conspicuous roles are to produce ATP through respiration, and to regulate cellular and energy metabolism [1]. Interestingly, mitochondria are the only organelles of human cells, besides the nucleus, that contain their own DNA (mtDNA). Human mtDNA is a 16,569-bp, double-stranded circular molecule that contains 37 genes, and recent studies have demonstrated a relationship between mtDNA variants and several human complex diseases. Specifically, common variants in mtDNA have been shown to contribute to a variety of neurological and metabolic diseases [1], including diabetes [2], [3], Alzheimer's [4], and Parkinson's [5] disease.

Obesity is a common and highly heritable disease in humans [6], whose prevalence has increased steadily in the US. If current trends continue, it is anticipated that over 50% of American adults will be obese by 2030, and that nearly 20% of total US health-care expenditures will be directed to obesity-associated diseases [7]. Obesity can be viewed as a disease of energy imbalance in which energy intake exceeds energy expenditure, resulting in excessive fat storage [8]. Given the essential function of mitochondria in energy metabolism, the potential contribution of mitochondrial dysfunction to cellular energy imbalances, metabolic disorders, and obesity, has been the subject of several recent studies [9], [10]. For example, Pietiläinen et al. [9] found that mitochondrial energy metabolism pathways were down-regulated in obese twins in a study of 14 pairs of obesity discordant twins. Wortman et al. [10] found a significant correlation between mitochondrial ATP production and age-related body mass index (BMI) by researching 172 children investigated for a suspected disorder of oxidative phosphorylation (OXPHOS). Moreover, recent population-based association studies investigating the relationship between common mtDNA variants and obesity found some nominally significant signals [11], suggesting that genetic variants in mtDNA might play an important role in susceptibility to obesity.

In this study, we performed genetic association tests to systematically examine the relationship between common mtDNA variants and obesity-related phenotypes, including body fat mass and BMI. We genotyped a total of 445 mitochondrial single nucleotide polymorphisms (mtSNPs) across the whole mitochondrial genome in a large sample of 2,286 unrelated Caucasian subjects. Of these 445 mtSNPs, 72 passed quality control, and these 72 mtSNPs were used to test for associations with BMI or body fat mass. Furthermore, since human mtDNA usually exists in haplogroup forms, we further classified subjects into nine common European haplogroups previously defined by Torroni et al. [12], based on specific mtSNPs throughout the mitochondrial genome. We then conducted association analyses between these haplogroups and BMI or body fat mass.

## Results

The basic characteristics of the study sample set are summarized in Table 1. We first tested for associations between 72 mtSNPs and BMI or body fat mass. We summarized the major association results with BMI and body fat mass in Table 2. Data for 6 mtSNPs are presented, five of which showed nominally significant levels (*P*<0.05) of association with BMI (Table 2). However, none of those remained significant after adjustment for multiple testing. Five mtSNPs showed nominally significant levels (*P*<0.05) of association with body fat mass (Table 2). After adjustment for multiple testing, two mtSNPs, *mt4823* and *mt8873* were still significant, with adjusted *P* values of 4.94×10^-3^ and 4.58×10^-2^, respectively. The minor allele C of *mt4823* and allele A of *mt8873* were both associated with reduced fat mass values with the effect size (β) estimated to be 3.52 and 3.18, respectively. These two mtSNPs also achieved nominally significant levels of association with BMI (Table 2). In order to validate our results, we randomly split the total sample in half, and re-performed association tests between *mt4823* and *mt8873* and body fat mass. These two mtSNPs remained consistently significant in both random samples (Sample 1: *P* = 3.12×10^-3^ for *mt4823*, *P* = 1.41×10^-2^ for *mt8873*; Sample 2, *P* = 7.46×10^-3^ for *mt4823*, *P* = 4.05×10^-2^ for *mt8873*).

**Table 1:** Basic characteristics of the study subjects.

	Total sample
Number	2,286
Gender (Males/Females)	558/1,727
Age (years)	51.37 (13.76)
Weight (kg)	75.27 (17.54)
Height (cm)	166.35 (8.47)
BMI (kg/m^2^)	27.14 (5.75)
Body fat mass (kg)	24.17 (10.63)

Note: Data are shown as mean (standard deviation, SD.).

**Table 2:** Major association results of mtDNA variants with BMI and body fat mass (*P*<0.05).

mtSNP	Gene	Alleles	Amino change	MAF	*P*_BMI_	*P*_adjusted_	β_BMI_	*P*_body fat mass_	*P*_adjusted_	β_body fat mass_
*mt4823*	*ND2*	C/A	Val -> Val	0.042	5.58×10^-3^	4.01×10^-1^	-1.36	**6.86×10^-5^**	**4.94×10^-3^**	-3.52
*mt8873*	*ATPase6*	A/G	Phe -> Leu	0.029	6.32×10^-3^	1.51×10^-1^	-1.48	**1.27×10^-3^**	**4.58×10^-2^**	-3.18
*mt16321*	*D-loop*	A/G	---	0.014	4.03×10^-3^	1.45×10^-1^	-2.90	4.13×10^-3^	9.91×10^-2^	-4.81
*mt16141*	*D-loop*	C/T	---	0.027	1.39×10^-2^	2.51×10^-1^	-1.72	5.25×10^-3^	9.45×10^-2^	-3.29
*mt12247*	*tRNA Ser*	C/G	---	0.035	2.46×10^-1^	1.00	-0.75	3.82×10^-2^	5.50×10^-1^	-2.10
*mt11300*	*ND4*	C/T	Thr -> Thr	0.077	2.91×10^-2^	4.19×10^-1^	-0.92	1.34×10^-1^	7.45×10^-1^	-1.21

For haplogroup analyses, all nine common European haplogroups were observed in our sample (Table 3). 95.7% of subjects belonged to these haplogroups, and the frequency of each haplogroup was consistent with the findings of previous studies of similar North American populations [5], [12] (Table 4). After association tests, one haplogroup (X) showed a strong signal with BMI (adjusted *P* = 8.31×10^-3^) and body fat mass (adjusted *P* = 5.67×10^-4^). Subjects classified as haplogroup X had significantly lower BMI and fat mass values compared to subjects who did not belong to haplogroup X, and the β was estimated to be 2.86 and 6.03, respectively. Considering the two significant SNPs we identified (mt4823 and mt8873), we further re-constructed haplogroup X including these two mtSNPs. The newly constructed haplogroup X captured by mt4823, mt8873, mt7029, mt10399 and mt14471, was in high linkage disequilibrium (LD) with the original haplogroup X (*r* ^2^ of 0.87). The newly constructed haplogroup X was also significantly associated with BMI and body fat mass, with adjusted *P* values of 1.02×10^-2^ and 8.51×10^-4^, respectively. In order to validate our results we, again, randomly split the total sample in half, and re-performed association tests between haplotype X, body fat mass, and BMI. Results in the divided sample were still significant for body fat mass (Sample 1: *P* = 2.56×10^-3^, Sample 2: *P* = 9.65×10^-3^). For BMI, the *P* value was significant in sample 1 (*P* = 4.23×10^-3^), and marginally significant in sample 2 (*P* = 5.58×10^-2^).

**Table 3:** Classification of common European haplogroups.

Haplogroup	C4217T	C7029T	A8252G	G10399A	C11720T	G12309A	A13709G	C14471T	G15219A	T16393C
H	---	C	---	A	---	---	---	---	---	---
I	---	T	A	G	---	---	G	---	---	---
J	---	T	---	G	---	---	A	---	---	---
K	---	T	---	G	---	G	G	---	---	---
T	C	T	G	A	---	---	G	---	---	---
U	---	T	---	A	---	G	---	---	---	---
V	T	T	G	A	C	---	---	---	A	C
W	---	T	A	A	---	A	G	---	---	---
X	---	T	---	A	---	---	---	C	---	---

**Table 4:** Association results of common European haplogroups with BMI and body fat mass.

Haplogroup	*P*_BMI_	*P*_ajusted_	β_BMI_	*P*_body fat mass_	*P*_ajusted_	β_body fat mass_	frequency
H	0.440	NS	-0.203	0.533	NS	-0.271	0.437
J	0.975	NS	-0.093	0.876	NS	0.120	0.086
U	0.434	NS	-0.307	0.608	NS	-0.279	0.164
K	0.412	NS	-0.484	0.984	NS	-0.017	0.059
I	0.803	NS	0.086	0.572	NS	0.806	0.027
W	0.279	NS	0.904	0.222	NS	2.140	0.023
T	0.391	NS	0.206	0.625	NS	0.353	0.099
X	**8.31×10^-4^**	**8.31×10^-3^**	-2.856	**5.67×10^-5^**	**5.67×10^-4^**	-6.031	0.010
V	0.682	NS	-0.289	0.625	NS	-0.446	0.052

## Discussion

In the current study, in which we examined associations between body fat mass or BMI with mtSNPs and mtDNA haplogroups, we identified two mtSNPs, *mt4823* and *mt8873*, that were significantly associated with body fat mass after adjusting for multiple testing. These two mtSNPs also achieved nominally significant levels for association with BMI, but no significant association was detected between mtSNPs and BMI after adjusting for multiple testing. Our results also demonstrated that haplogroup X was significantly associated with both with BMI and body fat mass.

Our findings regarding BMI are largely consistent with the results of Saxena et al. [11] who detected nominal signals of association between BMI and specific mtSNPs in mitochondrial genes, including *ND1*, *ND2*, *ND3* and *ATPase6*; after adjusting for multiple testing, these associations disappeared. Saxena et al. [11] also found a nominally significant association between haplogroup X and BMI (one-side *P* value is 0.049). Thus, the potential link between haplogroup X and body fat mass is quite intriguing.

Although BMI is widely used as an index to identify overweight individuals and define obesity due to its ease of measurement, BMI cannot always distinguish obese people from large framed people. Sometimes, genetic association tests might be underpowered to detect obesity related genes using BMI independently. While using both BMI and body fat mass as response variable one may observe more interesting signals. This view was also supported by other studies. For example, the *CTNNBL1* gene was recently implicated in the development of obesity in a genome-wide association study using both BMI (most significant SNP: *P* = 2.69×10^-7^) and body fat mass (*P* = 4.99×10^-8^) as obesity phenotypes [8], [13]. However, if BMI was investigated independently, the signal didn't achieve the genome-wide significance level (after multiple testing: *P* = 0.06). Therefore, BMI can be complemented by body fat mass, to comprehensively characterize obesity status.

The *mt8873* A/G SNP is a nonsynonymous SNP, that causes an amino acid substitution of Phenylalanine (Phe) to leucine (Leu). The *mt8873* SNP is located in the *ATPase6* gene (encodes ATP synthase F0 subunit 6), which is essential for ATP synthase function. ATPase6 is a key component of the proton channel, and plays a direct role in the translocation of protons across the mitochondrial membrane. Mutations in *ATPase6* have been implicated in several human diseases [14], [15], [16]. For example, two children found to carry an inherited mutation in the *ATPase6* gene suffered from mild encephalopathy, and became obese following a period of normal postnatal growth [17]. Using *Escherichia coli* as a model, Ogilvie et al. [18] found that missense mutations in the *ATPase6* gene can affect the ability of the enzyme to pump protons and synthesize ATP. Therefore, the mechanism by which *mt8873* A/G affects obesity risk might be through alterations in the function of ATP synthase.

The *mt4823* SNP is located in the NADH dehydrogenase 2 gene (*ND2*), which is involved in the respiratory chain system and belongs to complex I of the mitochondrion. The *mt4823* C/A is a synonymous change, which may act as a surrogate marker. The effect might be mediated through nearby SNPs in LD with *mt4823*. An additional explanation for the pathological mechanism of the *mt4823* is that *mt4823C* and *mt4823A* may differentially affect OXPHOS. However, the potential mechanisms by which these SNPs impact obesity related phenotypes must be confirmed through further functional analyses.

A potential limitation of our study is that our findings are still unsubstantiated due to a lack of replication in other independent large samples, especially for haplogroup X that has a low frequency (0.01). However, we have cross-validated our results in sub-samples established by randomly dividing our sample in half. Additional support for our findings come from the study of Saxena et al. [11], suggesting that our findings are certainly worthy of follow-up. We intend to recruit additional subjects so that we can perform replication analyses in an independent sample. Moreover, we are hopeful that publication of our findings will facilitate replication analyses by other groups.

In conclusion, we identified two mtSNPs (*mt4823* and *mt8873*) and mtDNA haplogroup X as markers that were significantly associated with reduced body fat mass. Our findings suggested that common variants in mitochondrial DNA play a role in variations of body fat mass. Further studies are warranted to explore the generality of our findings, and to determine the mechanisms underlying the risk of obesity.

## Materials and Methods

### Study Subjects

The study was approved by the local institutional review boards or the office of research administration of all participating institutions. After signing an informed consent form, all subjects completed a structured questionnaire addressing anthropometric variables, lifestyles, and medical history. The sample contained 2286 unrelated adults, who were randomly identified from our established and expanding database currently containing more than 10,000 subjects. All of the chosen subjects were Caucasians of Northern European origin living in the Midwestern US. They were normal healthy subjects defined by a comprehensive suite of exclusion criteria [19]. Briefly, subjects with chronic diseases and conditions involving vital organs (heart, lung, liver, kidney and brain) and severe endocrine, metabolic or nutritional diseases that might affect fat metabolism were excluded from this study.

BMI was calculated as body weight (in kilograms) divided by the square of height (in meters). Weight was measured in light indoor clothing without shoes, using a calibrated balance beam scale, and height was measured using a calibrated stadiometer. Body fat mass was measured with Hologic 4500 DEXA machines. The short-term reproducibility (coefficient of variation) of BMI and fat mass measurements is on average 0.2 and 1.1%, respectively. Measurement of body fat mass by DEXA is considered to be highly accurate and gold standard. The correlation between BMI and fat mass was 0.85 (*P*<0.01) in this sample.

### Genotyping and quality control

Genomic DNA was extracted from whole human blood using a commercial isolation kit (Gentra systems, Minneapolis, MN, USA) following the standard protocol. Genotyping was carried out using the Affymetrix Genome-Wide Human SNP Array 6.0 (Affymetrix, Santa Clara, CA, USA), which includes 445 mtSNPs throughout the mitochondrial genome, according to the Affymetrix protocol. Briefly, approximately 250 ng of genomic DNA was digested with restriction enzyme NspI and StyI. Digested DNA was adaptor-ligated and PCR-amplified for each sample. Fragment PCR products were then labeled with biotin, denatured, and hybridized to the arrays. Arrays were then washed and stained using Phycoerythrin on Affymetrix Fluidics Station, and scanned using the GeneChip Scanner 3000 7G to quantitate fluorescence intensities. Data management and analyses were conducted using the Genotyping Command Console. SNPs were identified using Birdsuite (version 1.5.2; http://www.broad.mit.edu/mpg/birdsuite/analysis.html).

In order to ensure high quality of the genotyping data, quality control procedures were conducted as follows. First, only samples with a minimum call rate of 95% were included. After repeated experiments, all samples (n = 2,286) met this criteria and the final mean call rate reached a high level of 98.93%. We also excluded individuals with hidden relatedness, which was generated from identity-by-descent matrix file at pi_hat >0.15 using PLINK[20]. Eighty-three pairs of individuals showed possible relatedness with pi_hat greater than 0.15. Thus, one of each pair was excluded from the subsequent analyses. Second, out of the initial 445 mtSNPs, we discarded mtSNPs: 1) with a call rate <95% (n = 25); 2) with genotyping concordance rate <95% (obtained by duplicate samples, n = 6); 3) with a minor allele frequency (MAF) <0.01 (n = 342; which was consistent with HapMap data in which 366 of 445 mtSNPs had a MAF less than 0.01). Because most mtSNPs were observed as rare SNPs (MAF <0.01) and were therefore excluded, only 72 common mtSNPs passed our quality control for subsequent analyses. The basic characteristics of these mtSNPs are summarized in Table S1.

### Statistical analyses

Before association analyses, principal component analysis implemented in EIGENSTRAT [21] was used to correct for potential population stratification that may lead to spurious association results. The first ten principal components emerging from the EIGENSTRAT analyses, along with sex, age and smoking status were included in the multiple regression model to tested for their correlations with BMI and fat mass. Significant variables (*P*≤0.05) were then included as covariates to adjust the raw BMI and fat mass values. The residuals after adjustment were used for association analyses.

#### Single-mtSNP Analysis

All association analyses were conducted in R v.2.11. For single-mtSNP variants, Student's t-test model was used to assess the difference in subjects carrying different mtSNP alleles. A raw *P* value of <0.05 in our study was considered nominally significant; this was further subjected to Benjamini and Hochberg's (BH) procedure [22] to account for multiple comparisons. The effect size (β, regression coefficient) was estimated by linear regression.

#### Haplogroup Analysis

According to the published references and the Mitomap database [3], [12], [23], we selected 10 specific mtSNPs to define the most common European haplogroups including H, I, J, K, T, U, V, W, and X (Table 3). Genotypes of these 10 mtSNPs were combined to construct mitochondrial haplogroups. Haplogroups that could not be assigned to one of these nine major haplogroups by mtSNP combinations were designated as “others”. For haplogroup association analyses, we determined the difference between phenotypic characteristics of each individual haplogroup and all other haplogroups pooled into one group. This is conceptually the same as the binary SNP allele comparison, which was accomplished by Student's t-test model. The effect size (β) was estimated by linear regression. Multiple testing was also adjusted by BH procedure [22].

For the significant mtSNPs or haplogroups we identified, we further split the total sample in half randomly, and re-performed the association tests in those two random samples. This approach enabled us to cross-validate our results, without having an independent sample.

## Supporting Information

Table S1 **Properties of SNPs in mtDNA tested in this study.**
(DOC)
