# Supplementary material for: Genetic Association Study of Common Mitochondrial Variants on Body Fat Mass
Source: PLoS One. 2011 Jun 29;6(6):e21595. doi: 10.1371/journal.pone.0021595 (PMC3126834; doi:10.1371/journal.pone.0021595)
Supplement: Table S1 — Properties of SNPs in mtDNA tested in this study. (DOC) [file pone.0021595.s001.doc]

Table S1. Properties of SNPs in mtDNA tested in this study

|  | mtSNP | Position | Minor Allele | Major Allele | MAF | Gene |
| --- | --- | --- | --- | --- | --- | --- |
|  | *mt348* | 348 | G | A | 0.024 | D-loop |
|  | *mt705* | 705 | G | T | 0.119 | 12S rRNA |
|  | *mt752* | 752 | A | G | 0.018 | 12S rRNA |
|  | *mt759* | 759 | G | T | 0.024 | 12S rRNA |
|  | *mt1440* | 1440 | A | G | 0.030 | 12S rRNA |
|  | *mt1702* | 1702 | G | A | 0.025 | 16S rRNA |
|  | *mt1813* | 1813 | G | A | 0.131 | 16S rRNA |
|  | *mt2708* | 2708 | A | G | 0.451 | 16S rRNA |
|  | *mt3012* | 3012 | A | G | 0.231 | 16S rRNA |
|  | *mt3198* | 3198 | C | T | 0.099 | 16S rRNA |
|  | *mt3506* | 3506 | C | T | 0.020 | ND1 |
|  | *mt3608* | 3608 | A | G | 0.011 | ND1 |
|  | *mt4217* | 4217 | C | T | 0.200 | ND1 |
|  | *mt4716* | 4716 | G | A | 0.032 | ND2 |
|  | *mt4823* | 4823 | C | A | 0.042 | ND2 |
|  | *mt5461* | 5461 | A | G | 0.029 | ND2 |
|  | *mt5496* | 5496 | G | A | 0.020 | ND2 |
|  | *mt6168* | 6168 | T | A | 0.013 | COI |
|  | *mt6186* | 6186 | G | A | 0.013 | COI |
|  | *mt7029* | 7029 | C | T | 0.447 | COI |
|  | *mt8252* | 8252 | A | G | 0.055 | COII |
|  | *mt8861* | 8861 | A | G | 0.013 | ATPase6 |
|  | *mt8873* | 8873 | A | G | 0.029 | ATPase6 |
|  | *mt9124* | 9124 | A | G | 0.017 | ATPase6 |
|  | *mt9699* | 9699 | G | A | 0.081 | COIII |
|  | *mt10239* | 10239 | C | T | 0.035 | ND3 |
|  | *mt10399* | 10399 | G | A | 0.193 | ND3 |
|  | *mt10464* | 10464 | G | A | 0.106 | tRNA Arg |
|  | *mt10551* | 10551 | T | C | 0.074 | ND4L |
|  | *mt10590* | 10590 | C | T | 0.010 | ND4L |
|  | *mt10591* | 10591 | C | A | 0.012 | ND4L |
|  | *mt10916* | 10916 | G | A | 0.011 | ND4 |
|  | *mt11147* | 11147 | A | G | 0.026 | ND4 |
|  | *mt11252* | 11252 | C | T | 0.197 | ND4 |
|  | *mt11300* | 11300 | C | T | 0.077 | ND4 |
|  | *mt11468* | 11468 | G | A | 0.227 | ND4 |
|  | *mt11675* | 11675 | T | C | 0.020 | ND4 |
|  | *mt11720* | 11720 | C | T | 0.497 | ND4 |
|  | *mt11813* | 11813 | C | T | 0.079 | ND4 |
|  | *mt11915* | 11915 | T | C | 0.025 | ND4 |
|  | *mt11948* | 11948 | G | A | 0.021 | ND4 |
|  | *mt12008* | 12008 | T | C | 0.016 | ND4 |
|  | *mt12247* | 12247 | C | G | 0.035 | tRNA Ser |
|  | *mt12309* | 12309 | G | A | 0.224 | tRNA Leu |
|  | *mt12502* | 12502 | T | C | 0.035 | ND5 |
|  | *mt12613* | 12613 | C | T | 0.092 | ND5 |
|  | *mt12634* | 12634 | A | C | 0.024 | ND5 |
|  | *mt12706* | 12706 | A | G | 0.075 | ND5 |
|  | *mt13106* | 13106 | C | T | 0.024 | ND5 |
|  | *mt13618* | 13618 | G | A | 0.093 | ND5 |
|  | *mt13709* | 13709 | A | G | 0.108 | ND5 |
|  | *mt14471* | 14471 | C | T | 0.016 | ND5 |
|  | *mt14794* | 14794 | G | A | 0.067 | Cytb |
|  | *mt14906* | 14906 | T | C | 0.104 | Cytb |
|  | *mt15044* | 15044 | T | C | 0.040 | Cytb |
|  | *mt15219* | 15219 | G | A | 0.046 | Cytb |
|  | *mt15302* | 15302 | T | C | 0.012 | Cytb |
|  | *mt15327* | 15327 | A | G | 0.012 | Cytb |
|  | *mt15453* | 15453 | T | G | 0.193 | Cytb |
|  | *mt15608* | 15608 | G | A | 0.104 | Cytb |
|  | *mt15785* | 15785 | G | A | 0.011 | Cytb |
|  | *mt15885* | 15885 | T | C | 0.020 | Cytb |
|  | *mt15925* | 15925 | G | A | 0.062 | tRNA Thr |
|  | *mt16094* | 16094 | G | A | 0.066 | D-loop |
|  | *mt16141* | 16141 | C | T | 0.027 | D-loop |
|  | *mt16173* | 16173 | C | T | 0.040 | D-loop |
|  | *mt16225* | 16225 | A | G | 0.075 | D-loop |
|  | *mt16258* | 16258 | T | C | 0.085 | D-loop |
|  | *mt16272* | 16272 | T | C | 0.090 | D-loop |
|  | *mt16313* | 16313 | C | T | 0.201 | D-loop |
|  | *mt16321* | 16321 | A | G | 0.014 | D-loop |
|  | *mt16521* | 16521 | T | C | 0.337 | D-loop |
